# Biocidal radiuses of abamectin, thiamethoxam and sulfoxaflor droplets controlling against wheat aphid (*Sitobion avenae*)

**DOI:** 10.1371/journal.pone.0205598

**Published:** 2018-11-08

**Authors:** Li Cui, Guobin Wang, Daibin Yang, Shahzad Ali Nahiyoon, Xiaojing Yan, Huizhu Yuan

**Affiliations:** Key Laboratory of Integrated Pest Management in Crops, Ministry of Agriculture, Institute of Plant Protection, Chinese Academy of Agricultural Sciences, Beijing, China; Fred Hutchinson Cancer Research Center, UNITED STATES

## Abstract

Spraying insecticide is a common practice in the control against pest insects. However, little attention has been paid to the biocidal radius of droplets. Therefore, in this study, we investigated the biocidal radiuses of abamectin, thiamethoxam and sulfoxaflor droplets controlling against wheat aphid (*Sitobion avenae*). The mortality of *S*. *avenae* showed a droplet density dependent process that can be described by an exponential model. Calculated mortality limit (*A*_2_) varied with the concentration of insecticides. Although similar LD_50_ values were observed in abamectin (13.77 ng aphid^−1^) and sulfoxaflor (14.52 ng aphid^−1^) against *S*. *avenae*, sulfoxaflor had a larger biocidal radius (r_50_) than abamectin due to its translocation ability at the same concentration. And sulfoxaflor had a relatively larger biocidal radius than thiamethoxam (LD_50_ = 68.42 ng aphid^−1^) because it is more toxic to *S*. *avenae*. The ratio of r_50_/VMD was introduced to estimate the potential of droplets. Droplets generated by the air atomizing nozzle (VMD = 43 μm) had higher value of r_50_/VMD than the centrifugal atomizing nozzle (VMD = 153 μm). Our results indicated that the mortality limit can be reached at a concentration of an insecticide. The biocidal radius of a droplet is different from its actual size. The LD_50_ and translocation ability of insecticides contributed to their biocidal radius. Ratio of r_50_/VMD is an indicator of droplets’ insecticidal potential. Smaller droplets generated by the air atomizing nozzle have higher insecticidal potential.

## Introduction

The English grain aphid, *Sitobion avenae* (Fab.), is the dominant pests of wheat and other cereal crops worldwide, causing most damage at the wheat filling stage. This sap-sucking insect can damage crops through direct feeding on phloem and xylem sap, secreting honeydew onto leaves that reduce the plants’ photosynthesis, and indirectly through transmission of viruses such as Barley Yellow Dwarf Virus [1]. Chemical control is currently the main programme for Integrated Pest Management (IPM) against this insect pest. Sulfoxaflor, a new sulfoximines insecticide is highly effective against a wide range of sap-feeding insects, especially against aphids. Like neonicotinoids, sulfoxaflor interacts with the nicotinic acetylcholine receptor (nAChR), but in a target site that is distinct from other insecticides [2–3]. Thiamethoxam is a neonicotinoid insecticide that shows higher toxicity against a number of sucking insect pests, including aphid, rice planthopper and mirid bug [4]. Abamectin belongs to the family of avermectins, a class of macrocyclic lactones produced by a soil actinomycete, *Streptomices avermitilis* [5]. It is proved to act on the gamma-aminobutyric acid (GABA) receptor/chloride ionophore complex and glutamate-gated chloride channel and has been used widely as an insecticidal, nematicidal and acaricidal agent [6].

Spraying insecticide is a common practice in the control against insect pests [7]. It has been proved that the effectiveness of insecticides are related with droplet deposit density, droplet size and droplet concentration [8–9]. Droplet deposit density is a primary factor that influencing the control efficacy of insecticides. High deposit densities of carbaryl were found to be more effective than low deposit densities in immobilizing oriental fruit moth larvae [10]. Similarly, Polles reported that high densities of small droplets showed higher control efficacy against tobacco budworm, *H*. *virescens* than low densities of large droplets (the same concentration) [9]. In addition, droplet size is as important as droplet density because it influences the amount of pesticide depositing onto the target [11]. It was believed that the efficacy of insecticides was negatively related to droplet size, because smaller droplet with a higher droplet density and concentration can easily contact and kill the target insect pests [8]. Himel suggested that small droplets in the 15–80 μm range would be beneficial for increasing the efficacy and 20 μm droplets of methyl parathion were the optimum against bollworms [12]. Similar finding was reported by Mboob that 20 μm was the most suitable size for spraying against glasshouse whitefly [13]. Mortality of *Plutella xylostella* larvae was increased by applying 36 μm droplet compared with 274 μm droplet of permethrin [14]. And Salyani et al. observed that the mortality of citrus rust mite *Phyllocoptruta oleivora* (Ashmead) decreased when the droplet size of abamectin was increased from 44 to 693 μm [15]. However, small droplets increased the risk of off-target drift, evaporation and rain wash-off compared with the large droplets [16,17]. Therefore, the spraying droplet size needs to be determined in order to achieve optimal control of a target pest. However, little attention has been paid to the biocidal radius of droplets. The concept of biocidal radius is different from the actual droplet size. The biocidal radius of droplets could indicate the insecticidal potential of droplets and it is more valuable in field application than droplet size.

Our study was carried out to determine the effect of spray parameters such as droplet size, droplet density and concentration of active ingredient on the efficacy of insecticides against *S*. *avenae* under laboratory condition. And the biocidal radiuses of three insecticides at different concentrations were also investigated. The results of this experiment will provide useful information for spraying application to increase the insecticides application efficiency and decrease the insecticides usage.

## Materials and methods

### Insects and insecticides

*S*. *avenae* were obtained from the laboratory of Institute of Plant Protection, Chinese Academy of Agricultural Sciences. This colony was grown on wheat plants (Aikang 58) without exposure to insecticides at 20 ± 2°C, 70 ± 10% relative humidity and 16: 8 (L:D) photoperiod. The wheat plants were grown in plastic tubes (10 seeds tube^−1^) in the greenhouse. Five days after seed germination, *S*. *avenae* were transferred to these wheat plants.

Sulfoxaflor (95.9%) was a gift from Dow AgroSciences Inc, America. Thiamethoxam (98%) was purchased from Jiangsu Gengyun Chemical Co. Ltd, China. Abamectin (95%) was purchased from Hebei Weiyuan Chemical Co. Ltd, China. Dimethyl sulfoxide (DMSO) and triton X-100 were purchased from Beijing Chemical Reagent Co. Ltd.

### Topical bioassays

Twenty adult aphids were placed on the wheat leaves sitting on the 1% agar media in the plastic tubs (7 cm diameter). The walls of the tub above the leaf were coated with fluon to prevent escape. The aphids were allowed to settle for an hour before being dosed individually with 0.3 μL acetone containing the technical grade insecticide to be tested using a micro applicator (Burkard Manufacturing Ltd, UK). A range of concentrations were applied for three insecticides. Control aphids were dosed with 0.3 μL acetone. Three replications were used for each treatment. The dosed aphids were incubated at 20 ± 2°C under a 16:8 h light: dark regime. The responses of aphids were assessed 24 h after treatment. Aphids were considered to be dead if they could not be higher to move when touched with a brush.

### Spray deposit assessment

An air atomizing nozzle (Burkard Manufacturing, England) and a centrifugal rotary atomization nozzle (Institute of Plant Protection, Chinese Academy of Agricultural Sciences) were used to generate spray streams. The air atomizing nozzle was installed in the potter spray tower. Different deposit densities on leaves were achieved by different spray volumes (10 μL, 20 μL, 50 μL, 100 μL, 200 μL and 400 μL). The stock solutions (20,000 mg L^-1^) were prepared by dissolving the technical grade insecticides in DMSO. The spray solution was prepared by diluting the stock solution of insecticide with an aqueous solution containing 0.05% (w/v) triton X-100. Water sensitive paper (WSP) was used to check the droplet density. The droplets number on WSP was counted by the scanner and the “Deposit Scan” software [18]. The droplet diameter was measured by DP-02 Laser Particle Size Analyzer (OMEC Instruments Co., Ltd, China). The centrifugal rotary atomization was installed on a spray shelf. The spraying height was 0.5 m. At a working voltage of 10 V, the measured flow rate was 200 mL min^-1^ and the rotating speed was 8000 r min^-1^. Desired deposit densities were achieved by different moving speeds of the spray shelf. The droplet diameter and the droplet density were detected according to the method mentioned above.

### Spray treatments

Spray targets consisted of wheat leaves infested with *S*. *avenae* and WSP. Wheat leaves containing about fifty *S*. *avenae* were used in the experiments described below. WSP and wheat leaves infested with *S*. *avenae* were put in the petri dishes and sprayed with thiamethoxam, abamectin and sulfoxaflor solutions. Afterwards each petri dish was covered with a perforated lid with fine mesh to provide ventilation. Then the petri dishes were stored in an incubator at 20 ± 2°C, 70 ± 20% RH and a 16:8 h light: dark photoperiod for 24 h until mortality was assessed. Control aphids feeding on aqueous triton X-100-treated wheat showed <10% mortality in all bioassays.

### Statistical analysis

The adjusted mortality was calculated using the Schneider–Orelli formula [19]. The LD_50_ (median lethal dose), LN_50_ (the number of droplets required per square centimeter to achieve 50% insect kill) and their 95% confidence intervals (CIs) of insecticides were calculated using statistical software DPS (v 7.05) (Refine Information Tech. Co. Ltd, Hangzhou, China). The term biocidal area (A_50_) has been used to describe the area around a droplet in which at least 50% of the insects are killed. It is equal to half the treated area divided by the LN_50_ value (median lethal number of droplets) (Hewitt and Meganasa 1993). Biocidal radius (r_50_) was calculated based on the formula $A_{50}=\pi {r_{50}}^{2}$.

## Results

### Toxicity of abamectin, thiamethoxam and sulfoxaflor against *S*. *avenae*

Using a micro-application method (applying insecticide directly to the dorsal surface of each aphid), the toxicities of three insecticides against *S*. *avenae* were shown in Table 1. These three insecticides have completely different LD_50_ values. The LD_50_ value of abamectin was 13.77 ng aphid^−1^ with its fiducial limit in the range of 11.28~16.80 ng aphid^−1^. The LD_50_ value of sulfoxaflor was 14.52 ng aphid^−1^ with its fiducial limit in the range of 11.54~18.26 ng aphid^−1^. Thiamethoxam is less toxic against *S*. *avenae*. Its LD_50_ value was 68.42 with its fiducial limit in the range of 37.22~125.76 ng aphid^−1^.

**Table 1:** The LD_50_ value of three insecticides against *S*. *avenae*.

Insecticides	LD_50_ (95% CI^a^) (ng aphid^−1^)	Regression equation	*r*
Abamectin	13.77 (11.28~16.80)	y = 1.9663x+2.7606	0.9815
Thiamethoxam	68.42 (37.22~125.76)	y = 1.2980x+2.6180	0.9415
Sulfoxaflor	14.52 (11.54~18.26)	y = 1.8582x+2.8411	0.9879

^a^ Confidence interval

### Spray droplet size

Volume median diameter (VMD) also known as Dv50 or Dv0.5, refers to the midpoint droplet size, where 50% of total volume of liquid sprayed drops with diameters larger than median value and 50% with smaller diameter. The VMD of droplets generated by the air atomizing nozzle and centrifugal rotary atomization nozzle was 43 ± 2 μm and 153 ± 5 μm, respectively.

### Droplet density dependent mortality of *S*. *avenae*

**Fig 1:**
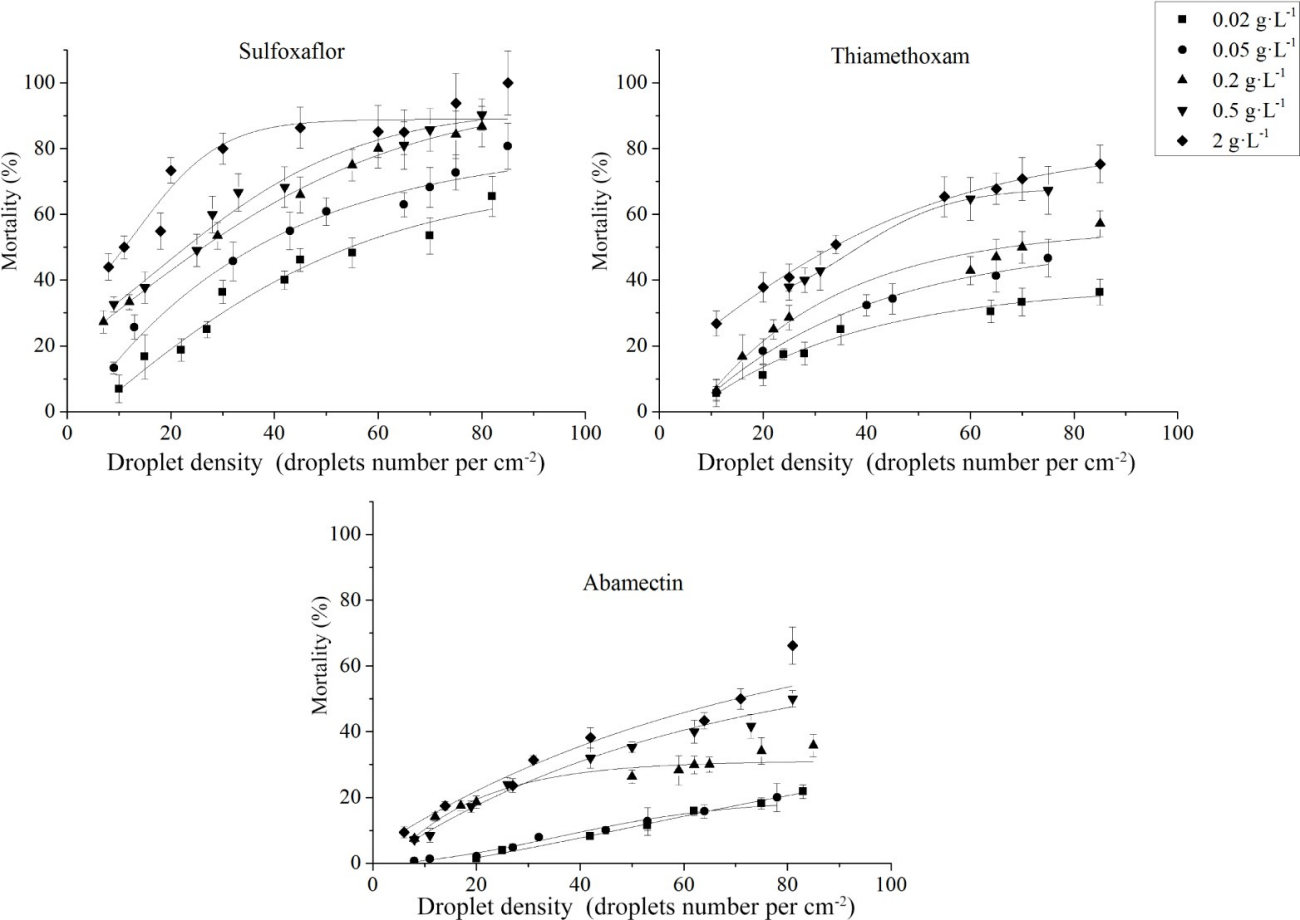
Relationship between droplet density and *S*. *avenae* mortality under different concentrations (0.02, 0.05, 0.2, 0.5, 2 g L^-1^) applied by the air atomizing nozzle (VMD = 43 μm).

**Fig 2:**
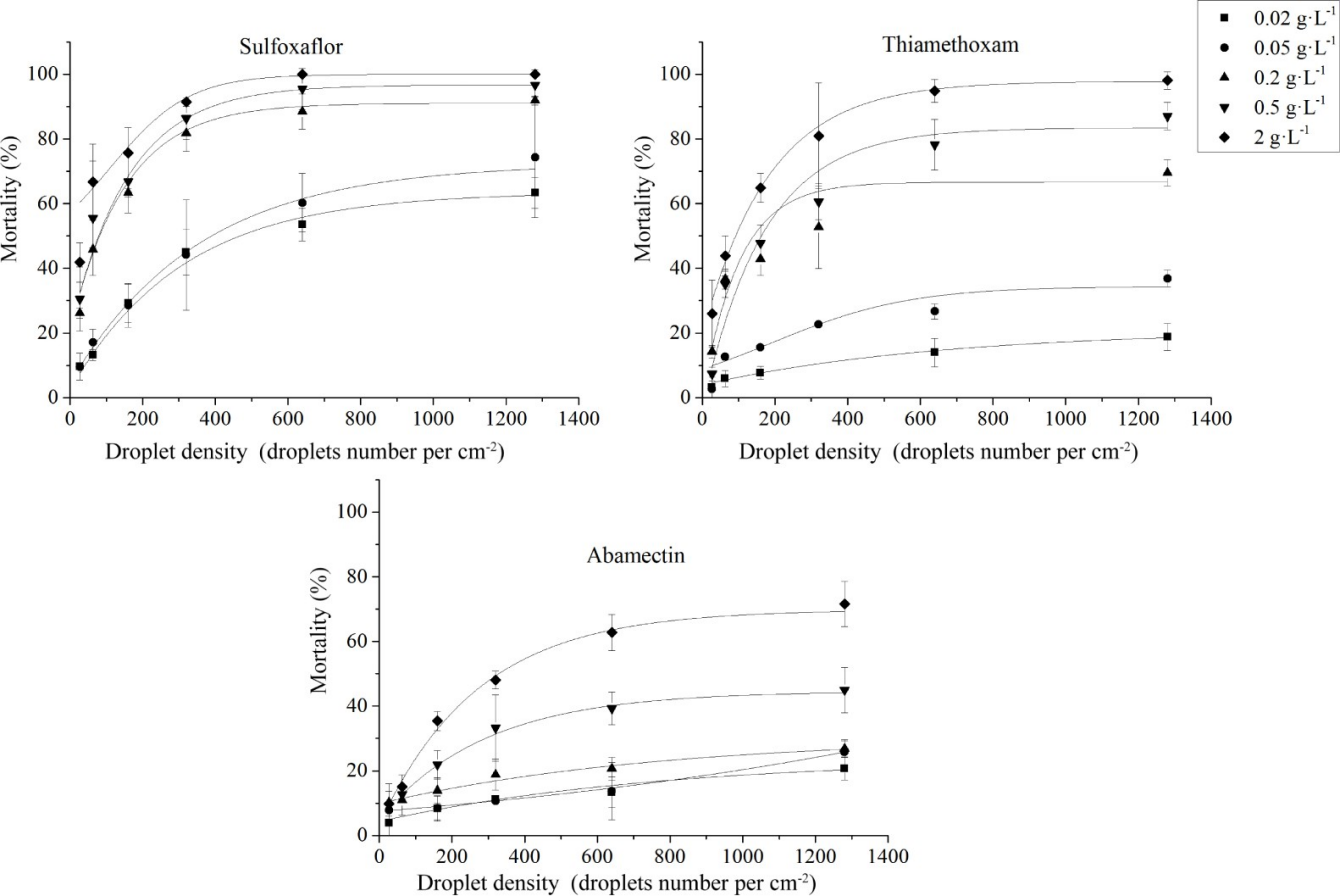
Relationship between droplet density and *S*. *avenae* mortality under different concentrations (0.02, 0.05, 0.2, 0.5, 2 g L^-1^) applied by the centrifugal atomizing nozzle (VMD = 153 μm).

Fig 1 showed the relationship between the mortality of *S*. *avenae* and droplet density generated by the air atomizing nozzle. The mortality of *S*. *avanae* increased with the increasing concentrations of three insecticides. It was observed that the mortality of *S*. *avanae* was gradually approaching a limit when the droplet density was getting higher and higher. Statistically, relationship between the mortality of *S*. *avenae* and droplet density can be described by the model

$$\text{y}=A_{1}+\frac{A_{2}-A_{1}}{1+10^{\left(LOGx0-x\right)p}}$$

where y is the mortality of *S*. *avanae*, x is the applied droplet density, *A*_2_ is the calculated mortality limit, *x*_95_ was the calculated droplet density to reach 95% of *A*_2_, and *A*_*1*_, *x*_*0*_, *p* are constants. The calculated mortality limit (*A*_2_) from fitting the model was shown in Table 2. It was shown the mortality limit also increased with the applied concentration of three insecticides. For thiamethoxam, the mortality limit increased from 21.14% at 0.02 g L^-1^ to 97.18% at 2 g L^-1^. For sulfoxaflor, the mortality limit increased from 62.75% at 0.02 g L^-1^ to 99.49% at 2 g L^-1^. But for abamectin, in the range of 0.02 to 2 g L^-1^, the mortality of *S*. *avanae* increased with the increasing droplet density and the mortality curve did not level off even at high droplet density. The mortality limit extrapolated from the applied model increased from 26.74~71.39%. The calculated *x*_95_ from the model data were also shown in Table 2. At the concentration of 0.5 g L^-1^, the value of *x*_95_ was 802.4, 666.1, 419.0 droplets cm^-2^ for abamectin, thiamethoxam and sulfoxaflor, respectively. At the concentration of 2 g L^-1^, the value of *x*_95_ was 845.7, 509.8, 357.5 droplets cm^-2^ for abamectin, thiamethoxam and sulfoxaflor, respectively. Similar results were observed in the centrifugal atomizing nozzle (VMD = 153 μm) (Fig 2).

**Table 2:** Mortality limit (*A*_2_) from fitting the model $\text{y}=A_{1}+\frac{A_{2}-A_{1}}{1+10^{\left(LOGx0-x\right)p}}$ to the data of *S*. *avenae* mortality and droplet density applied by the air atomizing nozzle.

Concentration (g L^-1^)	Mortality limit % (Mean ± SE)			Calc. x at 95% of *A*_*2*_ (droplets cm^-2^)		
	Abamectin	Thiamethoxam	Sulfoxaflor	Abamectin	Thiamethoxam	Sulfoxaflor
0.02	26.74 ± 6.97	21.14 ± 13.74	62.75 ± 3.99	—	—	847.1
0.05	27.65 ± 1.71	37.14 ± 12.51	77.50 ± 4.01	—	1231.9	1256.3
0.2	28.71 ± 9.87	68.29 ± 12.20	90.53 ± 3.09	—	586.0	416.0
0.5	44.71 ± 2.71	84.59 ± 10.70	96.15 ± 5.81	802.4	666.1	419.0
2	71.39 ± 3.80	97.18 ± 3.17	99.49 ± 5.83	845.7	509.8	357.5

### The LN_50_ and r_50_

The calculated LN_50_ and r_50_ of three insecticides sprayed by the air atomizing nozzle were shown in Table 3. For abamectin, when the concentration was < 0.5 g L^-1^, the mortality limit of *S*. *avanae* could not reach 50% and even the leaf surface was totally covered with abamectin solution, abamectin cannot kill 50% of tested *S*. *avanae*. Thus the calculated LN_50_ and r_50_ of abamectin were not available. This case also took place when the concentration of thiamethoxam was < 0.2 g L^-1^. In the range of 0.5 ~ 2 g L^-1^, the LN_50_ of abamectin decreased from 1505.6 droplets cm^-2^ to 373.0 droplets cm^-2^, and r_50_ increased from 0.10 mm to 0.21 mm. In the range of 0.2 ~ 2 g L^-1^, the LN_50_ of thiamethoxam decreased from 276.3 droplets cm^-2^ to 77.4 droplets cm^-2^, and r_50_ increased from 0.24 mm to 0.45 mm. For sulfoxaflor, the LN_50_ decreased from 539.4 droplets cm^-2^ to 36.7 droplets cm^-2^, and r_50_ increased from 0.17 mm to 0.66 mm in the range of 0.02 ~ 2 g L^-1^.

**Table 3:** The LN_50_ and r_50_ of three insecticides sprayed by the air atomizing nozzle (VMD = 43 μm).

Insecticide	Concentration (g L^-1^)	b ± SE	LN_50_ (95% CI^a^) droplets cm^-2^	r_50_ (mm)	r_50_/ VMD
Abamectin	0.02	—	>17,224	—	—
	0.05	—	>17,224	—	—
	0.2	—	>17,224	—	—
	0.5	0.79 ± 0.075	1505.6 (1057.1–2144.2)	0.10	2.33
	2	1.18 ± 0.059	373.0 (323.1–430.5)	0.21	4.88
Thiamethoxam	0.02	—	>17,224	—	—
	0.05	—	>17,224	—	—
	0.2	0.87 ± 0.13	276.3 (179.6–425.3)	0.24	5.58
	0.5	1.43 ± 0.14	188.6 (146.3–243.0)	0.29	6.74
	2	1.65 ± 0.094	77.4 (64.5–92.9)	0.45	10.47
Sulfoxaflor	0.02	1.05 ± 0.071	539.4 (434.1–670.3)	0.17	3.95
	0.05	1.18 ± 0.049	398.3 (353.1–449.3)	0.20	4.65
	0.2	1.26 ± 0.074	79.1 (65.6–95.4)	0.45	10.47
	0.5	1.46 ± 0.11	59.0 (45.1–77.3)	0.52	12.09
	2	1.36 ± 0.20	36.7 (25.0–54.0)	0.66	15.32

^a^ Confidence interval

When the droplets were generated by centrifugal atomizing nozzle, very similar situation occurred (Table 4). When the concentration of abmectin was < 0.5 g L^-1^ and the concentration of thiamethoxam < 0.05 g L^-1^, the mortality limit of *S*. *avanae* was lower than 50%. In the range of 0.5 ~ 2 g L^-1^, the LN_50_ of abamectin decreased from 90.5 droplets cm^-2^ to 67.0 droplets cm^-2^, and r_50_ increased from 0.42 mm to 0.49 mm. In the range of 0.05 ~ 2 g L^-1^, the LN_50_ of thiamethoxam decreased from 80.2 droplets cm^-2^ to 31.4 droplets cm^-2^, and r_50_ increased from 0.45 mm to 0.71 mm. For sulfoxaflor, the LN_50_ decreased from 55.3 droplets cm^-2^ to 10.8 droplets cm^-2^, and r_50_ increased from 0.54 mm to 1.21 mm in the range of 0.02 ~ 2 g L^-1^.

**Table 4:** The LN_50_ and r_50_ of three insecticides sprayed by the centrifugal atomizing nozzle (VMD = 153 μm).

Insecticides	Concentration (g L^-1^)	b ± SE	LN_50_ (95% CI^a^) droplets cm^-2^	r_50_ (mm)	r_50_/ VMD
Abamectin	0.02	—	>1360	—	—
	0.05	—	>1360	—	—
	0.2	—	>1360	—	—
	0.5	1.42 ± 0.054	90.5 (82.1–99.7)	0.42	2.75
	2	1.36 ± 0.16	67.0 (51.8–86.6)	0.49	3.20
Thiamethoxam	0.02	—	>1360	—	—
	0.05	1.70 ± 0.13	80.2 (68.2–94.3)	0.45	2.94
	0.2	1.65 ± 0.14	67.8 (57.3–80.3)	0.48	3.14
	0.5	1.70 ± 0.097	38.7 (36.9–40.7)	0.64	4.18
	2	1.51 ± 0.061	31.4 (29.7–33.1)	0.71	4.64
Sulfoxaflor	0.02	1.92 ± 0.12	55.3 (50.0–61.1)	0.54	3.53
	0.05	1.78 ± 0.11	35.2 (32.1–38.5)	0.67	4.38
	0.2	1.64 ± 0.13	20.2 (17.3–23.7)	0.89	5.82
	0.5	1.87 ± 0.16	19.8 (17.1–23.0)	0.90	5.88
	2	1.79 ± 0.18	10.8 (8.9–13.2)	1.21	7.91

^a^ Confidence interval

### The ratio of r_50_/VMD

The ratio of r_50_/VMD was introduced to estimate the insecticidal potential of droplets. The ratios of r_50_/VMD of three insecticides sprayed by the air atomizing nozzle were shown in Table 3. The value of r_50_/VMD was 4.88 when abamectin at the concentration of 2 g L^-1^. For thiamethoxam, the value of r_50_/VMD increased from 5.58 to 10.47 in the range of 0.2 ~ 2 g L^-1^. In the range of 0.02 ~ 2 g L^-1^, the value of r_50_/VMD increased from 3.95 to 15.32 for sulfoxaflor. When the droplets were generated by centrifugal atomizing nozzle, the ratios of r_50_/VMD were shown in Table 4. In the range of 0.5 ~ 2 g L^-1^, the value of r_50_/VMD increased from 2.75 to 3.20 for abamectin. For thiamethoxam, the value of r_50_/VMD increased from 2.94 to 4.64 in the range of 0.05 ~ 2 g L^-1^. For sulfoxaflor, the value of r_50_/VMD increased from 3.53 to 7.91 in the range of 0.02 ~ 2 g L^-1^. So smaller droplets generated by the air atomizing nozzle have higher insecticidal potential.

## Discussion

Generally, the mortality of *S*. *avanae* increased with the increasing droplet density of three insecticides. But for thiamethoxam and sulfoxaflor, the mortality-droplet density curve leveled off when relative high concentration was employed (Fig 1). This result demonstrated there exists a mortality limit at a concentration of the spray dilution, and the mortality limit varied with different concentrations. For example, when sulfoxaflor was sprayed by the air atomizing nozzle at a concentration of 0.2 g L^-1^, the mortality limit can reach 90.53%, whereas at 0.02 g L^-1^, the mortality limit decreased to 62.75%. According to the calculated mortality limit in Table 2, the values of mortality limit were far below 50% if a relative low concentration was applied. For example, the mortality limit was only 21.24% when thiamethoxam was sprayed at the concentration of 0.02 g L^-1^. This efficacy cannot satisfy the requirement of agricultural practice. Therefore, to achieve desired efficacy against the pest insects, calculating the minimal insecticide concentration and droplet density is essential. For example, the minimal concentration of thiamethoxam was 2 g L^-1^ and the minimal droplet density was 509.8 droplets cm^-2^ for the control of wheat aphid with an air atomizing nozzle (VMD = 43 μm). The minimal concentration of sulfoxaflor was 0.2 g L^-1^ and the minimal droplet density was 416.0 droplets cm^-2^. At these concentrations and droplet densities, the control efficacy was very close to the mortality limit and the mortality could meet the control requirement. However, the abamectin treatments at tested concentrations cannot meet the practical requirement because the recorded mortality and calculated mortality were < 72%.

Many studies have demonstrated that spray droplet size is an important factor [20]. People established the theory of biological optimum droplet size when different kinds of pesticides were sprayed onto the target surface [21–22]. Himel et al. proved that the maximum diameter for efficient insecticide spray droplets is less than 50 μm [12]. However, the theory of biological optimum droplet size refers to the actual droplet size. Our results indicated biocidal radius is different from the actual droplet size. Whenever the droplets were generated by an air atomizing nozzle or a centrifugal atomizing nozzle, the biocidal radius increased with the increasing concentration of insecticides. If the concentration of insecticides was relatively low, the biocide radius was less than the actual droplet size, whereas the biocide radius was larger than the actual droplet size if a relatively high concentration was applied. For example, when thiamethoxam was sprayed by the air atomizing nozzle at the concentration of 0.02 and 0.05 g L^-1^, the r_50_ was less than its droplet size (VMD = 43 μm), whereas at 2 g L^-1^, the r_50_ was 10.47 times than the droplet size. Two reasons can be proposed to explain the difference. One is that individual pesticide droplet could moves out from each deposit to exert an influence beyond their boundaries and establish a biocidal area [23–25]. Adamas et al. reported that each deposit of permethrin is surrounded by a region where there is a high probability of mortality for whitefly larvae *Trialeurodes vaporariorum*, even though these sedentary insects have not been contacted by direct impact of insecticide [26]. And the overall control achieved on a treated surface depends on the summed effect of all the biocidal areas on that surface [27]. Another is that during the gradual evaporation, the concentration of droplet leftover would be higher and higher so the active ingredients would not distribute evenly across the droplet leftover.

The toxicity (LD_50_) of insecticide also contributes to the biocidal radius of an insecticide. The LD_50_ of thiamethoxam was lower than sulfoxaflor, and it exhibited relatively smaller biocidal radiuses than sulfoxaflor. Furthermore, the biocidal radius was related to the translocation ability of insecticides within the plant tissues. The LD_50_ of abamectin and sulfoxaflor is 13.77 and 14.52 ng aphid^−1^, respectively. But the biocidal radius of sulfoxaflor was 2 ~ 3 times that of the abamectin when compared at the same dilution concentration. The relatively larger biocidal radius of sulfoxaflor was due to its systemic activity, whereas abamectin cannot be translocated within plant tissues. This translocation made sulfoxaflor move further within plant tissues besides the above mentioned diffusion outside the deposit boundaries. Similarily, thiamethoxam showed larger biocidal radius than abamectin due to its systemic activity though the LD_50_ of thiamethoxam was lower than abamectin. Most research has been conducted to investigate the optimal nozzle, spraying pressure and droplet size on efficacy of insecticides against various insect pests. Unfortunately, the effect of the biocidal radius of the droplets was ignored when the droplet size was selected to spray an insecticide [21–22]. Our results indicated the biocidal radius can help spray workers improve the efficacy. The minimal concentration and the minimal droplet density of the insecticides should be taken into consideration.

Ratio of r_50_/VMD implicates the potential of droplets. A higher value of r_50_/VMD means the insecticidal activity of active ingredients can reach a relatively wider area. Thus the value of r_50_/VMD can be an indicator of droplets’ insecticidal potential at a concentration. For abamectin, the value of r_50_/VMD of the air atomizing nozzle was 1.5 times that of the centrifugal atomizing nozzle at 2.0 g/L. For thiamethox, the value of r_50_/VMD of the air atomizing nozzle was 1.6 ~ 2.3 times that of the centrifugal atomizing nozzle when compared at same concentration. For sulfoxaflor, the value of r_50_/VMD of the air atomizing nozzle was 1.1 ~ 2.1 times that of the centrifugal atomizing nozzle when compared at same concentration. Therefore, smaller droplets generated by the air atomizing nozzle have higher insecticidal potential.

Our results suggest that there was a large increase in mortality as droplet density increased, whereas the mortality limit can be measured at a concentration of an insecticide. The minimal concentrations and droplet density of three insecticides for controlling against wheat aphids should be considered in agricultural practice. The LD_50_ and translocation property of insecticides can affect their biocidal radius so sulfoxaflor had a larger biocidal radius than thiamethoxam and abamectin for the control of wheat aphids at same concentration. Ratio of r_50_/VMD is the indicator of droplets’ insecticidal potential. Smaller droplets generated by the air atomizing nozzle have higher insecticidal potential. However, wheat aphids are sucking insect pests. The biocidal radius of insecticides against the biting insect pests should be studied. And the field testing should be required for further validation.
